# A Literature Review of Barriers and Opportunities Presented by Digitally Enhanced Practical Skill Teaching and Learning in Health Science Education

**DOI:** 10.1080/10872981.2022.2068210

**Published:** 2022-04-21

**Authors:** Cuisle Forde, Annie OBrien

**Affiliations:** Discipline of Physiotherapy, School of Medicine, Trinity College Dublin, the University of Dublin, Dublin, Ireland

**Keywords:** Blended, flipped classroom, practical, online, skill

## Abstract

**Introduction:**

An evidence gap exists identifying the challenges and opportunities presented by digitally enhanced practical skill teaching and learning in health science education. A literature review was carried out to address this gap and to provide recommendations for overcoming identified challenges.

**Method:**

A systematic search strategy was carried out using PRISMA guidelines. The research databases PubMed, ERIC, Medline and CINHL, were searched using MeSH terms. Barriers and opportunities were identified through deductive thematic analysis of the included articles.

**Results:**

Of the 602 articles identified through the database screening, 29 were included in the current review. Potential challenges posed by technologically supported practical skill teaching identified were i) Inaccessibility and Inequity of Online Learning (ii) Digital illiteracy Among Staff (iii) Technological Challenges (iv) Lack of Engagement with Preparatory Material Hinders Practical Learning (v) Lack of Staff–Student Interaction (vi) Negative Attitudes Towards Online Learning and (vii) Skill Suitability. The opportunities presented by digital technologies identified were (i) Facilitates Higher Order Learning (ii) Ability to Practice in a Safe Environment (iii) Efficacious Use of Class Time (iv) Access to Education (v) Learning Brought to Life (vi) Diverse Range of Learning Materials (vii) Promotes Autonomous Learning.

**Discussion:**

This literature review demonstrates the acceptability and usability of digitally enhanced practical teaching in health science education among students and educators.

**Conclusion:**

To consolidate the positive disturbances in higher education from the Covid-19 pandemic, potential barriers to online delivery and student engagement must be acknowledged and addressed by relevant stakeholders. Recommendations detailed as part of this paper suggest means of overcoming barriers and leveraging opportunities.

## Introduction

Developing practical skill competency is a significant component of health science education programmes. This encompasses, but is not limited to, conducting laboratory tests, skills in communication, observing and taking physiological measurements, and practicing correct techniques when physically assessing, handling, moving, or providing treatment to patients. Rapid advancements in technology over the past number of decades [1] has seen a rise in the use of digital technologies to enhance teaching [2]. In its simplest form, this includes the digitisation of teaching content (i.e. PowerPoint presentations, the use of virtual teaching platforms, e.g. Blackboard), whilst also expanding to more complex forms of technological integration involving virtual realities and digitally enhanced teaching props [2]. In this paper, digitally enhanced practical skill teaching will be defined as any use of technology, both simple and complex, to support the acquisition of a practical skill.

Educators commonly introduce technology into the classroom in the form of blended learning, or through a flipped-classroom approach [3], while students routinely engage with websites, recorded videos, and podcasts to complement and enhance classroom teaching [4]. Recent years have further seen the integration of advanced technologies (i.e. virtual simulations of clinical scenarios) in the classroom gaining popularity, presenting students with a unique opportunity to consolidate practical skill learning through digitally enhanced clinical practice.

The use of technology in higher education became a necessity from March 2020 when Coronavirus disease 2019 (Covid-19) was first declared a global pandemic [5]. Under the guidance of public health officials, global emergency lockdowns resulted in university and business closure; social distancing measures were introduced to reduce the spread of the virus, resulting in suspension of face-to-face teaching and a pivot to online learning. This abrupt shift in teaching methods presented novel challenges for educators in the health sciences [6], who were required to adjust practical skills teaching for online delivery.

Globally, educators adopted creative and innovative methods to online practical skills teaching, which one may not intuitively consider suited to distance learning. Staff utilised technology to design and develop materials for practical skills teaching to overcome the pedagogical challenges posed by the Covid-19 pandemic. Pre-recorded instructional videos, narrated PowerPoint presentations, and live practical classes with students practicing at home are examples of digitally enhanced teaching approaches adopted by health science educators to teach practical skills remotely [7–9].

Research studies have predominantly focused on the efficacy and acceptability of digital technology in teaching theoretical knowledge and practical skills, specifically, in surgical [9–11], dental [12,13], nursing [14–16] and physiotherapy education [4,17,18]. However, there is a gap in the literature exploring the barriers and opportunities presented by digital technologies in health science education generally, and in practical skill acquisition specifically.

The available evidence suggests that digitally enhanced teaching is a promising pedagogical approach to practical skill teaching in a post-Covid era [6,14]. Thus, education in the health sciences is presented with a unique opportunity to consolidate the positive disturbances from the Covid-19 pandemic and to improve staff teaching and student learning post-pandemic; specifically, practical teaching and learning may benefit from technological integration [19]. Understanding the institution-level, educator-level, and student-level challenges to technological integration in health science education could enhance student engagement and improve learning outcomes [4]. However, it is equally important to formulate recommendations to successfully overcome these challenges. Therefore, this literature review aims to identify the barriers, and opportunities presented by digital technologies in teaching and learning practical skills within health science education to address this gap in the literature. It also aims to provide recommendations to overcome these challenges, leverage opportunities, and promote successful integration of digitally enhanced practical teaching in future health science education.

## Methods

### Inclusion and Exclusion Criteria

A systematic search strategy was carried out using the Preferred Reporting Items for Systematic Reviews and Meta-Analyses (PRISMA) guidelines [20]. Studies reporting on opportunities and barriers to online practical skill teaching in health science education were identified. A strict list of inclusion and exclusion criteria was developed with the support of the PICO framework. The following inclusion criteria were applied: (i) articles were published in English (ii) between Jan 2000 and October 2021 (iii) were peer-reviewed (iv) participants were university students or academic staff within the health sciences (v) learning or teaching practical skills using digital technologies (vi) explored skill acquisition, clinical competency, or attitudes towards e-learning experience. Articles were excluded if they (i) focused on a non-student or staff population, (ii) did not explore practical skill acquisition using digital technologies, (iii) did not explore barriers or opportunities presented by digital technologies and (iv) the full text was unavailable. Opinion pieces and articles excluding research data were omitted.

### Search Strategy

The search was first conducted on 30 August 2021 and was carried out for 10 weeks; the final search conducted on 12 October 2021. Due to the rapid advancements in technology, and the introduction of technology assisted teaching materials in classrooms in the 21^st^ century [1], the literature was searched between 1 January 2000 and 12 October 2021. MeSH terms, subject terms and synonyms were identified using the EBSCOhost thesaurus. The search strategy utilised Boolean operators (AND, OR) and truncations. Using the EBSCOhost research platform, the electronic databases MEDLINE, ERIC, and CINAHL Complete were searched using subject terms (Table 1). The individual database PubMed was searched using MeSH terms (Supplementary material). A complementary search of the reference list of key papers was also carried out.

**Table 1.** Search strategy for EBSCO Host using subject terms (SU)

[SU Education, Distance OR ‘Computer Assisted Instruction’ OR TI (‘“Blended Learning”’ OR ‘“Computer Uses in Education”’ OR ‘“Distance Education”’ OR ‘“Educational Technology”’ OR ‘“Electronic-Learning”’ OR ‘“Web Based Instruction”’ OR ‘online learning’ OR ‘e-learning’ OR ‘“distance education”’ OR ‘“distance learning”’ OR ‘flipped classroom’ OR ‘remote learning’ OR ‘“computer based training”’ OR “‘online instruction’ OR ‘“virtual learning”’ OR ‘“web-based learning”’ OR ‘“technology-enhanced learning”’ OR ‘educational technology’)] AND [SU ‘Medical Education’ OR TI (‘“Medical Education”’ OR ‘“Allied Health Occupations Education”’ OR ‘“Health Science*”’ OR ‘“Medical School”’ OR ‘“Medical Student*”’ OR physiotherap* OR nurs* OR dent* OR surgical OR “‘health education’“)] AND [SU Clinical Competence OR TI (‘“Skill Development”’ OR ‘“Skill* Training”’ OR ‘practical skill*’ OR ‘clinical skill*’ or ‘skill acquisition’ or ‘skill development’ or ‘skill learning’ OR ‘“Clinical Experience”’ OR ‘surgical skill*’)] AND [SU (‘Barrier* and Facilitator*’ OR ‘Teacher Attitudes’ OR ‘Student Attitudes’ OR ‘Facilitators (Individuals)’ OR Barriers OR Opportunities OR ‘Student Satisfaction’) OR TI (‘Barriers and Facilitators’ OR ‘Student Attitudes’ OR ‘Student Teacher Attitudes’ OR ‘Teacher Attitudes’ OR ‘student satisfaction’ OR ‘Perception*’) OR AB (‘Barriers and Facilitators’ OR ‘Student Attitudes’ OR ‘Student Teacher Attitudes’ OR ‘Teacher Attitudes’ OR ‘student satisfaction’ OR ‘Perception*’)]

### Screening Process

An initial search of the databases revealed a total of 602 papers. The papers were exported to Endnote and a strict screening of the title and abstract was carried out by the author. Papers that were deemed potentially suitable for the review following the title and abstract screening were downloaded for full-text screening.

**Figure 1.**
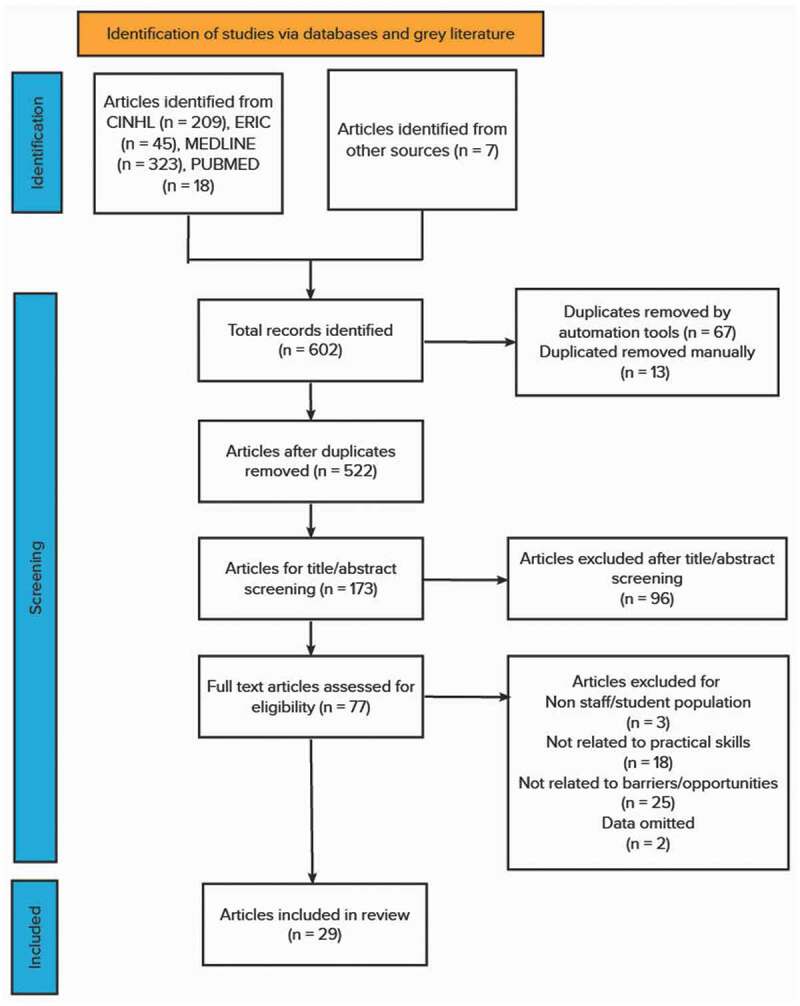
PRISMA flowchart of the screening process.

During full-text screening, the following data was chartered: author, year, title, journal, aim, participants and sample size, intervention, skill, outcome measures and results, barriers, and opportunities. A PRISMA flow chart [21] of the search process and outcome is illustrated in Figure 1. Following the screening process, 29 articles were deemed suitable for inclusion in the literature review (Table 1). A summary of excluded papers is available upon request.

### Analysis

**Figure 2.**
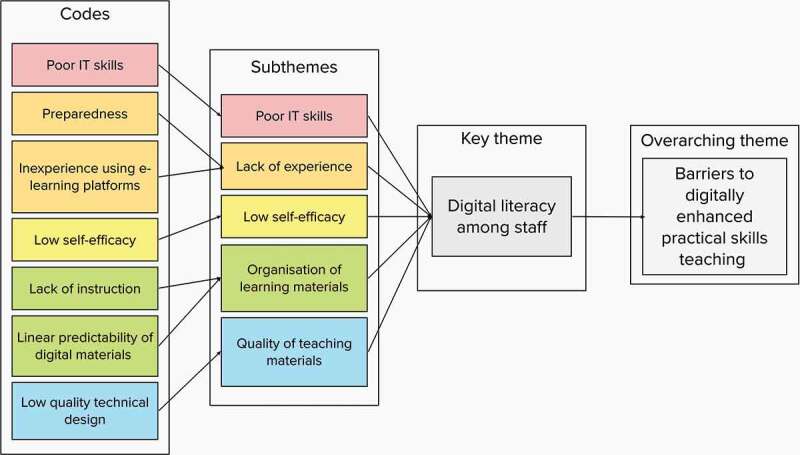
Example of visual mapping of codes for generating themes.

Thematic analysis using a deductive orientation [22] was conducted on the included studies. This approach was employed because of its flexibility [23] and suitability in synthesising mixed research designs [24]. Data familiarisation was achieved by reading and re-reading articles. During the systematic coding process, qualitative and quantitative data was tagged with a code label; similar codes were clustered together to identify coherent patterns in the data. These clusters were colour coded and mapped under two overarching themes (barriers and opportunities). An example of this mapping process is illustrated in Figure 2. Preliminary candidate themes [25] were generated and continuously reviewed and refined during the reflective process. Candidate themes that did not have adequate data to support them were either eliminated or combined with similar preliminary themes [22] until 7 key themes per overarching theme were consolidated. Thematic overlapping was difficult to avoid due to the multifaceted nature of themes, however, consolidation of key themes ensured meaningful framing of the data [22].

When formulating the recommendations, a process was followed which primarily considered the evidence collected. This was then further informed and influenced by the authors’ lived experience in the area of online education in health science. An effort was made to ensure recommendations were applicable to a breadth of pedagogical situations reflecting the diversity of health science educational needs. The generalisability of recommendations can be seen as a strength of this research, which strives to be mindful of the diversity in those who teach, those who learn and the resources they engage with. Examples given alongside recommendations enable them to be more specific and actionable.

## Results

### Study Characteristics

Overall, 29 studies were included in the literature review, ranging in publication years from 2007 to 2021. Most were quantitative (n = 13, 45%), followed by mixed methods (n = 10, 35%) and qualitative (n = 3, 10%), while 3 were reviews (10%). Studies were carried out in Australia, Europe, Asia, North and South America, and Africa. Further descriptives are detailed in Table 2.

**Table 2.** Summary of Included Articles

Reference Number	Author, Year (Country)	Aim	Participants	Skill	Data Collection Tools
**6**	Pei & Wu, 2019 (USA, UK, Spain, Brazil, Germany, China, Iran, Indonesia, India)	Exploring the efficacy of online learning in meeting learning outcomes compared to traditional teaching	Undergraduate medical students	Knowledge and skills	Systematic review and meta-analysis
**7**	Gamage et al., 2020 (Global)	Review of online teaching and lab practices pre-Covid and student experiences	Students and staff generally	General skills teaching	Review
**10**	McGann et al., 2021 (USA)	Explore the feasibility and efficacy of an online lab in teaching surgical skills	3rd and 4th yr medical students (N = 86)	Identification of instruments, knot-tying and suturing	Survey of student knowledge, skill acquisition and competency
**19**	Björklund & Silén, 2021 (Sweden)	Explore student learning of interprofessional communication while working collaboratively with a virtual patient	Occupational therapy and physiotherapy students (N = 8)	Interprofessional communication	Recorded and transcribed student conversations and non-verbal communication
**27**	Dhar et al., 2021	Literature review of virtual reality implementation, and student experiences, in medical training	Medical students	General practical and clinical skills, communication and physical examination	Review
**28**	Sebbani et al., 2021 (Morocco)	Explore student attitudes towards online learning	Health science students (N = 111)	General clinical skills	Questionnaire of student attitudes
**29**	Muflih et al., 2021 (Jordan)	Explore students’ attitudes towards online learning	Medical students (N = 1210)	General clinical skills and core competencies	Survey of student attitudes
**30**	Bdair, 2021 (Saudi Arabia)	Investigate student and staff perspectives of distance learning	Nursing students (N = 10) and staff (N = 10)	Psychomotor skills and core competencies	Semi-structured interviews
**31**	Bloomfield & Jones, 2013 (UK)	Attitudes and experiences of blended learning approach to clinical skills training	Postgraduate nursing students (N = 83)	Hand decontamination, oral medication administration, injection technique, physical hygiene, feeding a patient, aseptic technique, elimination needs, skin assessment and pressure area care	Questionnaire and focus groups for feedback on student perception and experience
**32**	Abbasi et al., 2020 (UAE, USA, UK, Australia, Canada, Egypt, Iraq, Malaysia, Nigeria, Pakistan, Saudi)	Explore student attitudes and satisfaction towards online learning during Covid-19	Health science students (N = 1255)	General clinical and technical skills	Questionnaire on attitudes, perceptions, satisfaction
**33**	Abdelaziz et al., 2011 (Egypt)	Evaluate the effectiveness of an online learning programme against traditional teaching	2nd year nursing students (N = 276)	Central venous pressure and electrocardiogram procedures	Questionnaire on knowledge, attitudes, opinions. Checklist on procedural steps
**34**	Rodriques-Fernandes et al., 2020 (Global)	Systematic review of digital microscopy as a learning and assessment tool	Medical and dental students	Identify and differentiate clinical features, diagnoses of specimens	Objective measures of student achievements, perceptions
**35**	Woodham et al., 2015 (UK)	Inform the development and use of virtual patients	Medical students (N = 119), teaching staff (N = 18)	Clinical reasoning skills	Surveys of student/staff perceptions
**36**	Reinhart et al., 2021 (Germany)	Explore student and staff attitudes and experiences with online learning	5th yr medical students (N = 16) and teaching staff (N = 8)	Patient history taking, physical examination	Focus group discussions
**37**	Lehmann et al., 2013 (Germany)	Explore student and staff attitudes towards blended learning using virtual patients for skills training preparation	5th yr medical students (N = 617)	Lumbar puncture, paediatric life support, bladder puncture	Student clinical examination, staff interviews
**38**	Londgren et al., 2021 (Europe and North America)	Investigate the efficacy of the FC approach in preparing students for clinical skill lab	Veterinary educators (N = 101)	General clinical skills	Survey of educator attitudes
**39**	Choi et al., 2021 (South Korea)	Evaluate the feasibility of using FC to provide respiratory system assessment content and explore student-centred learning pre- and post-FC	2nd yr nursing students (N = 91)	Respiratory system assessment	Questionnaire with open-ended questions
**40**	Ali & John, 2019 (Bahrain)	Efficacy of online videos in for clinical skill competency and satisfaction	Nursing students (N = 26)	Hand washing, surgical bed making and nasogastric tube deeding	Competency skill evaluation checklist, satisfaction questionnaire, and focus group discussion.
**41**	Jang & Kim, 2014 (South Korea)	Explore student experience and attitudes towards using online videos for learning clinical skills	Medical students (N = 411)	Unspecified clinical skills	Semi-structured interviews and questionnaires of student attitudes
**43**	Zamberg, Schiffer, & Stoermann-Chopard, 2021 (Switzerland)	Explore student perceptions of and satisfaction with an online learning module in renal semiology	2nd yr medical students (N = 105)	Renal semiology/nephrological semiology	Survey with open-ended questions. Video conferencing call to gather feedback on student perceptions and satisfaction
**44**	Posey & Pintz, 2017 (USA)	Explore student and faculty perceptions of a blended learning experience and student digital literacy skills	Nursing students (N = 36) and staff (N = 6)	Communication skills, collaboratively building patient cases, digital literacy	Student surveys of attitudes, pre- and post-program assessment of digital literacy, staff interviews
**45**	Dolan, Hancock & Wareing, 2015 (UK)	Evaluate the efficacy of delivering an ECG skills laboratory using online and traditional learning approaches	1st yr health science students (N = 22)	ECG electrode placement and interpretation	MCQ and practical examination
**46**	Badowski, Rosslet & Reiland, 2021 (USA)	Examine student attitudes towards use of traditional clinical and manikin-based simulation experiences to meet learning outcomes	Nursing students (N = 97)	Communication skills	Clinical Learning Environment Comparison Survey
**47**	Wotton et al., 2010 (Australia)	Evaluate student experiences with high-fidelity simulations	3rd yr nursing students (N = 300)	General clinical skills	Evaluation form with open questions
**48**	Schoening, Sittner & Todd, 2006 (USA)	Explore student attitudes towards a pre-term labour patient simulation compared to traditional teaching	Nursing students (N = 60)	Perform physical assessment, develop a care plan, critical thinking, delivery of preterm infant	Survey of learning objectives, open box comment for student perceptions
**49**	Reilly & Spratt, 2007 (Australia)	Student and staff perceptions and experiences of high-fidelity simulation to prepare for clinical practice.	2nd yr nursing students (N = 41) and teaching staff (amount unspecified)	Patient care, communication skills	Survey and focus group discussion of perceptions
**50**	Kleinert et al., 2007 (USA)	Improve student self-efficacy and decrease perceived difficulty of treating children with developmental disorders	Dental students (N = 51)	Communication skills	Pre- and post- knowledge test, usability scale
**51**	Wise, McIvor, & Mangione, 2016 (USA)	Assess student usage and perceived value in using web-based simulation in medical education	Medical students (N = 138)	Clinical use of pulmonary artery catheters	Survey response of student perception
**52**	Arslan et al., 2018 (Turkey)	Student satisfaction with watching educational videos pre- or post- practical skills lab	Nursing students (N = 213)	Wound care	Questionnaire on satisfaction

Thematic analysis identified seven key themes for each of the two overarching themes: (i) Barriers to digitally enhanced practical skills teaching and (ii) Opportunities presented by digitally enhanced practical skills teaching in health science education.

Barriers to digitally enhanced practical skills teaching:

- Theme 1.Inaccessibility and Inequity of Online Learning
- Theme 2.Digital Literacy Among Staff
- Theme 3.Technological Challenges
- Theme 4.Lack of Engagement with Preparatory Material Hinders Practical Learning
- Theme 5.Lack of Staff–Student Interaction
- Theme 6.Negative Attitudes Towards Online Learning
- Theme 7.Skill Suitability

Recommendations to overcome these barriers are suggested.

Opportunities presented by digitally enhanced practical skills teaching:

- Theme 1.Facilitates Higher Order Learning
- Theme 2.Ability to Practice in a Safe Environment
- Theme 3.Efficacious Use of Class Time
- Theme 4.Access to Education
- Theme 5.Learning Brought to Life
- Theme 6.Diverse Range of Learning Materials
- Theme 7.Promotes Autonomous Learning

Table 3 maps recommendations to themes. 77

**Table 3.** Mapping recommendations to barrier and opportunity themes

Recommendation	Barrier theme	Opportunity theme
1 Provide physical and technological supports	1. Inaccessibility and Inequity of Online Learning 3. Technological Challenges 6. Negative Attitudes Towards Online Learning	3. Efficacious Use of Class Time 4. Access to Education
2 Support the continued professional development of those who teach and share resources	1. Inaccessibility and Inequity of Online Learning 2. Digital Literacy Among Staff 3. Technological Challenges 5. Lack of Staff-Student Interaction 6. Negative Attitudes Towards Online Learning	4. Access to Education
3 Collaborate with software developers as key stakeholders	2. Digital Literacy Among Staff 3. Technological Challenges 5. Lack of Staff-Student Interaction 7. Skill Suitability	6. Diverse Range of Learning Materials
4 Develop procedures to promote high quality effective online education	3. Technological Challenges 4. Lack of Engagement with Preparatory Material Hinders Practical Learning 5. Lack of Staff-Student Interaction 6. Negative Attitudes Towards Online Learning	1. Facilitates Higher Order Learning 3. Efficacious Use of Class Time 6. Diverse Range of Learning Materials 7. Promotes Autonomous Learning
5 Make full use of online tools and possibilities	1. Inaccessibility and Inequity of Online Learning 4. Lack of Engagement with Preparatory Material Hinders Practical Learning 5. Lack of Staff-Student Interaction 6. Negative Attitudes Towards Online Learning	1. Facilitates Higher Order Learning 3. Efficacious Use of Class Time 4. Access to Education 5. Learning Brought to Life 6. Diverse Range of Learning Materials 7. Promotes Autonomous Learning
6 Seek feedback	3. Technological Challenges 5. Lack of Staff-Student Interaction 6. Negative Attitudes Towards Online Learning 7. Skill Suitability	

### Barriers to digitally enhanced practical skills teaching

#### Theme 1: Inaccessibility and Inequity of Online Learning

Ensuring accessibility and equity of digitally enhanced education has been identified as a significant challenge [26]. There are socioeconomic and environmental factors that reduce accessibility to online learning [27], such as unsuitable home environments [28], the financial demand on students to have essential learning equipment [6,29,30] and poor internet access [28,29].

Internet access is an ethical concern to policymakers and stakeholders [28], as it is a significant factor influencing access to online learning. During the Covid-19 pandemic, students reported lower satisfaction and greater negative experiences with digitally enhanced teaching in developing countries (i.e. Egypt, Nigeria and Iraq) when compared to students in developed countries (i.e. UK, USA and Australia) [31]. Students living in rural areas can also be disadvantaged compared to students based in cities as slow internet speed [29] and unreliable internet access [28] can present as challenges.

Lack of access to the internet and to technological devices required for online learning (i.e. laptops and webcams) places a financial burden on students and their families [6,29]. Focus group discussions revealed students experience stress and worry at the possibility of falling behind in their course due to lack of access to technological devices [30]. Of the 276 Egyptian nursing students learning to perform an electrocardiogram through either traditional teaching (N = 186) or an online module (N = 90), almost 75% of students lacked access to manikins during the in-person practical class, hindering their learning [32]. Additionally, institutions also face a financial burden when it comes to purchasing software licences and designing interactive and immersive learning materials for practical skill teaching, for example, augmented realities [26] and digital imaging software [33].

#### Theme 2: Digital Literacy Among Staff

A common barrier to learning reported by staff and students was educators’ lack of confidence, familiarity, and experience with digitally enhanced teaching methods [27]. This can result in low-quality design and delivery of learning materials, hindering student interaction, engagement, and overall learning. As practical skill teaching often focuses on visual, tactile and auditory information [34], poor quality of online audio and/or visual resources can be frustrating for learners [35].

The design, development and delivery of high-quality learning resources plays a significant role in supporting students to achieve the desired learning outcomes. Staff have described difficulties using digitally supported teaching methods, while being expected and required to become ‘experts’ [7] in the rapidly advancing technological methods that are supplementing and substituting traditional teaching approaches.

While a lack of instruction [28] incoherent or disorganised layout of online learning material [30] and difficulty identifying key information [35] are described as frustrating aspects of online learning, poor organisation of learning materials can also hinder student learning success [36]. For example, the linear progression of prognosis in virtual patients is a design element that can be criticised by students, due to a lack of comparability to a learning environment with real patients. These predictable patient progressions can be perceived as limiting opportunities for learning [37]. Alongside poor design, low visual and audio quality, and poor acting skills, have been cited as reasons preventing students from achieving desired learning outcomes during skill acquisition supported by online or digital tools [35].

#### Theme 3: Technological Challenges

Technological and logistical problems are proclaimed as a barrier to online learning throughout the academic literature. These issues include periodic unavailability of e-learning platforms [27], internet connectivity issues resulting in video buffering [38] and broken web links to resources [35]. When practicing communication and assessment skills with patients through video, an unstable internet connection can disrupt flow and rapport building [36]. Patient unfamiliarity or discomfort using technology can also impede skill acquisition [36].

Students can also experience technical problems when required to utilise large software packages to support their learning. For example, digital imaging software for remote laboratory skill acquisition can demand large quantities of data and high-speed connectivity [33]. This can present challenges for students owning basic-grade devices with limited storage space.

There are also technological challenges to assessment of practical skill acquisition online. A lack of standardised methods for assessing clinical skills online has been described as ‘concerning’ [7]. Developing remote assessment methods may present challenges to educators, who must ensure students practice academic integrity during remote assessment periods [29]. Furthermore, unreliability of internet connectivity and technical issues across online learning platforms can cause additional stress for students during the assessment period [29].

#### Theme 4: Lack of Engagement with Preparatory Material Hinders Practical Learning

Another obstacle to practical skill acquisition is a lack of student engagement with essential learning material prior to an in-person practical class, challenging the efficacy of the flipped-classroom approach to teaching. When students arrive to practical skills classes without engaging with the preparatory online materials, there is a discrepancy in student ability from the class onset [38]. This issue raises safety concerns, while placing an increased burden on the students who come to the class prepared, as well as on educators who must revisit the preparatory materials with unprepared students [38]. Lack of pre-class preparation results in a slower class pace, hinders critical discussion, and challenges learning opportunities [30,39].

The flipped classroom approach can also result in students struggling with the responsibility of self-directed study [39]. A hybrid health assessment module required nursing students to engage with video and text-based learning materials prior to a practical skills class [39]. The students reported difficulty engaging with preparatory materials independently, lacked confidence in their understanding of key information, and required educators to place a greater emphasis on the importance of engaging with the pre-class material [39]. Health-care students also reported difficulty with time-management and organisation [36], low motivation for engaging with online learning materials, and difficulty with attention when studying remotely during Covid-19 [28,29]. Research suggests that postgraduate students tend to be highly self-motivated and more likely to engage with online resources for independent study when compared to undergraduate students [6].

#### Theme 5: Lack of Staff–Student Interaction

Students report a lack of interaction with teaching staff as a challenge when learning theory and practical skills remotely [28,29,36,40]. When watching video demonstration of clinical skills, students feared misinterpreting key information due to the inability to ask questions and clarify information in the absence of teaching staff [40,41]. Students experienced feelings of isolation when learning remotely and found it difficult to form friendships with limited peer interaction [30].

Staff also described lack of interaction with students as a disadvantage to theoretical and practical teaching online. When teaching through video-conferencing platforms, there is a lack of visual and verbal feedback to indicate student engagement and understanding [36]. Educators described the lack of feedback typically gained through non-visual cues when teaching as ‘very difficult’ [36].

Online learning has been criticised for the reduced opportunity for real-time feedback when practicing clinical skills [10]. Online multiple-choice questionnaires (MCQs) can deliver immediate feedback [42], however, MCQs are limited to assessing knowledge-based acquisition rather than skill [10]. Assessing skill acquisition remotely poses challenges; innovative modifications to assessment techniques are required to ensure learning outcomes are met [7]. When assessing clinical skills remotely, 60% of staff reported pre-recorded videos hindered their ability to provide accurate feedback as they would in real time [10], while students reported a preference for skill demonstration in real-time [9] and found staff-feedback more beneficial than peer-feedback [10].

#### Theme 6: Negative Attitudes Towards Online Learning

Learning practical skills using the support of digital technologies is often perceived as an inadequate substitute to hands-on learning [29,30]. Patient interaction is deemed essential by students [28]. Although online learning was satisfactory for theoretical teaching [30], teaching staff and nursing students believed learning practical skills and competencies online was an ‘inappropriate’ replacement for traditional hands-on learning [29,30]. Of 1,328 health-care students, approximately 75% reported practical learning was best suited to clinical or laboratory environments and felt ill-prepared tending to patients without in-person practical skills sessions [31]. Medical students believed in-person practical learning alongside an experienced doctor was invaluable [43]. Lack of experience with online learning may also drive negative attitudes [30,43].

Negative attitudes have also been influenced by negative perceptions, and past negative experiences of online teaching and learning [35,43,44] which can hinder engagement with technologically supported teaching methods [28]. Students are typically required to take responsibility for their learning and engage with learning material independently, leading to a common misconception among students that educators are ‘not doing their job’ [44]. Students have thus been found to perceive this pedagogical approach as time-intensive [28,39]; staff, however, also report an increased workload due to the time-consuming preparation and recording of online learning materials [44].

#### Theme 7: Skill Suitability

It is important to consider the clinical attributes of a skill when appraising its suitability for online delivery; specifically, complex skills (e.g. requiring haptic awareness) may be best suited to traditional teaching approaches [34].

One study that illustrates this theme involved a module on ECG electrode placement and interpretation, which was delivered to undergraduate health science students, in-person or online. When assessed on theoretical learnings with an MCQ, both groups performed comparatively, however the traditional learning group performed significantly better in practical skill acquisition (i.e. ECG electrode placement) [34]. The authors concluded that the skill’s complexity deemed it unsuitable for online-learning as the e-learning group were disadvantaged in their limited use of sight and sound, while the traditional learning group benefited from kinaesthetic learning and real-time feedback [34].

Communicating with patients and obtaining a medical history was another practical skill that students struggled with in the pivot to remote learning during the Covid-19 pandemic [36]. The overall experience of taking a patient’s medical history over webcam was described as ‘more strenuous’, with increased importance placed on the wording of questions posed to patients to ensure compassionate communication [36]. Teaching staff identified this technological teaching method as limiting, with alternative methods potentially required for patients with disabilities unable to communicate via webcam [36].

### Opportunities Presented by Digitally enhanced Practical Skills Teaching

#### Theme 1: Facilitates Higher Order Learning

Theoretical and practical teaching using the support of digital technologies can promote deep learning through critical thinking and transference of knowledge [36]. Nursing students who engaged in a flipped-classroom approach for a health assessment module found the in-person classroom discussion was useful for supporting higher order learning in the form of collaborative problem solving [39]. Blended approaches to practical skills teaching have been described as valuable due to the opportunities to study preparatory materials in one’s own time, with class time maximised for critical discussion of learning materials while listening and learning from multiple perspectives [39,44].

When designed well, simulated patients and clinical environments have been shown to support students in developing critical thinking skills as they interact with a realistic learning scenario [45]. Students are required to think on their feet and make decisions quickly [46], while anticipating and responding to varied patient outcomes [45]. Bjorkland et al. [18] conducted a study exploring interprofessional communication skills between physiotherapy and occupational therapy students while working collaboratively with a virtual patient. Students engaged in interprofessional knowledge transference that encouraged them to develop a holistic view of the virtual patient’s presenting problem and view the patient’s presentation from multiple perspectives in future clinical practice [18]. When 300 nursing students were surveyed on their experience with three patient simulations, 92.4% reported the experience as suitably challenging [46]. Student action – and inaction – with the virtual patient has realistic consequences that staff believe may be more useful than a clinical scenario where student observation and engagement can be limited [47]. This method may also empower students or facilitate their autonomy as clinicians.

#### Theme 2: Ability to Practice in a Safe Environment

Online learning materials can help students safely familiarise themselves with a clinical or laboratory setting prior to real-life exposure, helping them to develop an awareness of potential hazards. The ability to revisit and replay visual demonstrations can also enhance understanding of each procedural step [29] and promote safe practice [7]. Digitised teaching tools can demonstrate what students ‘should be aiming for’ as they are not always guaranteed to observe best practice in a clinical setting [30].

Digital tools can also present students with a safe environment to practice prior to clinical exposure [48]. When practicing patient care with technologically enhanced manikins, students appreciated being able to exercise their learning and safely make mistakes [48] [48]. Many graduate nurses have reported difficulty in integrating and applying theoretical learning and clinical practice when required to act quickly in clinical scenarios [47]. However, nursing students described reduced anxiety and greater self-awareness when provided with an opportunity to practice their theoretical learning with a manikin [32]. For example, a virtual simulation of a preterm labour provided nursing students with a unique opportunity to critically appraise an emerging situation as it progressed and make decisions without the fear of real-life consequences [47]

Digital technologies can also provide students with opportunities not readily available or accessible in clinical environments to build skill competency. For example, dentistry students were provided with a unique opportunity to develop communication skills when interacting with a virtual patient specifically designed to mimic a young child with an intellectual disability [49]. The virtual patient was designed and developed with input from the parents of a child with Down syndrome, as well as with educators, to ensure realism. Following interaction with the virtual patient using a video-based simulation, students’ perceived difficulty of handling a similar situation in real-life was significantly reduced. Students also demonstrated significant gains in knowledge pertaining to communication and demonstration skills and reported a need for this type of educational tool in dentistry [49].

#### Theme 3: Efficacious Use of Class Time

A blended teaching approach can improve opportunities for student practical skill learning through efficacious use of in-person class time [38]. When asked about flipped classroom teaching [38] staff described improved use of class time with increased time afforded to providing support to struggling students, practicing challenging components of a skill, perfecting technique, and receiving personalised feedback from teaching staff [38]. Furthermore, students describe coming to class motivated to apply their theoretical learning to a practical setting [39]. Staff perceive this motivation directly resulting from the availability of interactive online learning materials for self-preparation, promoting student self-efficacy when in the learning environment [37].

Teaching practical skills online during the Covid-19 pandemic also enabled teaching staff to maximise scheduled teaching time [36]. Practitioner educators described a newfound appreciation for scheduled clinical skills labs compared to teaching in a demanding clinical environment [36]. Having time scheduled with patients and students for online teaching allowed practitioners to focus on student learning for the allocated time and avoid hurried and/or disrupted teaching sessions experienced in clinical environments [36].

#### Theme 4: Access to Education

The pivot to online learning has been deemed a facilitator of inequity due to high cost of electronic devices and internet access [26,28–30]. Despite this, online learning increases accessibility of education independent of student or staff location [36] and to a large demographic of students [34,47], particularly to students who may have previously been disadvantaged. When learning clinical skills through online video demonstrations, undergraduate medical students identified videos as a tool that enhances accessibility of education [41]. Students experienced challenges when watching in-person demonstrations, such as missing key visual information, whereas videos facilitated high-quality observation of skills in practice [41]. Additionally, accessing education online may help to address institutional concerns of increasing class sizes [47] high student–staff ratio [47] lack of student accommodation, and reduced availability of clinical placements [47].

Some students reported improved psychosocial health when learning remotely during the Covid-19 pandemic [27]. Balancing class time with clinical training pre-pandemic was described by students as negatively impacting concentration for theoretical learning [27]. Studying from home allowed students to approach learning from a relaxing and comfortable environment, at a time that was convenient to them, reducing learning fatigue and burnout [27,50]. Students and staff also describe reduced commute costs and time as an added benefit of online learning [27,43].

#### Theme 5: Learning Brought to Life

Digitally enhanced practical teaching has been described as an enriching and valuable learning experience by students [26,47] with an ability to bring learning to life [35]. Physical models, cadavers and volunteers provide limited learning opportunities when compared to well-designed digitally enhanced simulations that can range subtly, or drastically, in their clinical presentation and medical progression [26]. A review of augmented reality in medical training described anatomy programmes that demonstrated realistic actions and reactions of complex muscular systems, for example, eye movement, contextualising and animating theoretical learning [26]. Virtual reality also provided unique learning opportunities for medical students through an immersive learning experience that allows the learner to ‘interact with digital anatomical representation at all angles’ [26].

Virtual simulations can enable students to observe and engage with a virtual learning experience that mimics realistic and varied patient outcomes [48], facilitating application of theoretical learning to clinical scenarios [46–48]. Nursing students learning patient care and communication skills with simulated patients described the experience as helpful [46,48]. Simulations can help students visualise patient progression and the relationship between symptoms [46], enhance understanding of the patient–practitioner relationship [48], and increase confidence in application of theory to practice [46,48].

#### Theme 6: Diverse Range of Learning Materials

Using technology to teach practical skills can be used to facilitate Universal Design of Learning. Digital technology can provide educators with a unique opportunity to use varied teaching approaches [29] to accommodate varied learning styles and enhance the learning experiences for diverse types of learners [51]. Specifically, availability of various online learning materials allows students to engage with resources in a way that is meaningful to them [38]. Health science students have described visual learning aids, such as images and videos, as advantageous when learning clinical skills [30,37]. Technology enables students to critically interpret visual information through side-by-side comparison of digitised images [33] and has been described by students as a learning tool that increases their confidence when recalling information [30]. Digitally enhanced learning materials can also support skills teaching that relies heaving on auditory information, such as auscultation [30] as it allows for enhancement of audio information.

#### Theme 7: Promotes Autonomous Learning

Online learning places a focus on self-directed study, with students required to take responsibility for their own learning [29], taking time to become comfortable with a skill by rewinding, and revisiting skill demonstrative videos at their own convenience [37,51]. When working collaboratively with a virtual simulation patient, occupational therapy and physiotherapy students could work at their own pace, which they noted as a valuable component of the learning experience [18]. In a study exploring student nurse skill competency following self-paced interactive video recordings, students reported increased self-efficacy as a result of the flexibility afforded to their learning [40].

When preparatory materials are online for engagement prior to clinical skills classes, students must develop skills in organisation and time management to reach learning objectives [44]. Thus, technology facilitate students becoming active agents in their learning, moving away from the ‘sage on the stage’ phenomenon involving passive learning [30,44].

## Recommendations

### Provide physical and technological supports

Providing students with access to computer classrooms and the library in the evenings and weekends may support students who lack access to required software, technological devices, or stable internet connection at home. Investing in dedicated spaces for online learning would enable students to move between online and face-to-face teaching with greater ease, maximising teaching and learning time, without which students may have to commute home to adequately engage with online material in an appropriate learning space.

IT support for both students and staff is required to tackle any unforeseen or immediate issues related to teaching and learning [27,28], such as connectivity issues during an online exam [29]. Standard instructions and trouble-shooting guides should be developed for both students and staff to encourage continuity and clarity as it relates to technological issues. Creating a checklist for students prior to engaging in a live online exam could help overcome some common difficulties, for example, students can be reminded to ensure that their computers are fully charged and connected to a reliable source of internet. A means of contacting a member of staff during an exam should be clearly outlined to students and a staff member assigned to each exam in the same way an invigilator is present to assist during face-to-face exams.

Specific to the acquisition of practical skills, where possible students should be provided with access to equipment needed to practice skills in their own time (e.g. stethoscope and zinc-oxide tape).

### Support the continued professional development of those who teach and share resources

Facilitating staff with continuous professional development [52], in designing, developing, and delivering high-quality digital resources may enhance staff self-efficacy [53,54]. Promoting digital design skills enable educators to develop course content that students can meaningfully interact with [42,54], enhancing student engagement with the course curriculum [7,42], and overall promoting staff and student success.

Where appropriate, sharing of online educational resources should be encouraged. The creation of open educational resources (OERs) and supporting those developing teaching resources to use creative commons licences will enable more widespread use of high-quality relevant teaching material and enable those with limited resources to reap the benefits of online education, tackling the barrier of inequality. Many courses share learning outcomes but do not share teaching and learning materials. The numerous reasons for this include geographical and time constraints- both of which are potentially overcome with online learning. Where those who teach are supported to create high-quality online educational material, the ability to share that material should be addressed at an early stage in resource development. Using creative commons licencing will enable others to learn from, and if appropriate, use parts of or all of resources created by others, maximising the impact of online teaching and avoiding duplication of work. The creation and widespread use of high-quality OERs in this field could also help those who teach and those who learn in institutions without the means to develop learning resources (for example, those who teach in developing countries) and should be developed, within reason, with this in mind.

### Collaborate with software developers as key stakeholders

Greater collaboration between those who develop teaching tools (e.g. software designers and developers), and those who use them may help ensure relevant and user-friendly features, leading to improved outcomes for digitally enhanced education. Communicating teaching and learning needs to stakeholders involved in the development of digitally enhanced teaching tools may also result in the creation of new tools to overcome current shortfalls in this area and open further opportunities for practical skill teaching in the health sciences.

### Develop procedures to promote high quality effective online education

The development of minimum standards for online teaching material could improve the learner experience by ensuring variables such as sound and video quality are appropriate for the end user. This is arguably more important when online material is used to support the acquisition of practical skills, many of which require visual or auditory input. Ensuring a minimum standard for learning resources could positively influence learning experiences and decrease the negative perception many students hold towards online learning. For example, audio checks should be encouraged before streaming or recording teaching material and before attending live lectures. Although these features exist on most platforms, they are not routinely used which can lead to poor outcomes for the end user.

To enhance engagement, institutions could promote policies outlining expectations regarding online education, such as students turning their cameras on during class.

When preparatory work is essential it may be necessary to enforce completion of relevant online learning materials prior to arriving to class. In the context of blended practical learning, including consequences for incompletion [50], such as assigning marks to the online material that go towards student overall grades, could improve student interaction with preparatory materials. This may combat safety concerns and maximise the efficacy of in-person class time, ensuring that students reach higher levels of learning when engaging in face-to-face practical classes. It is not uncommon in higher level education for marks to be awarded for attendance or participation. The equivalent online would be for marks to be awarded for interaction and engagement with online resources. Typically, online education platforms enable data on student engagement to be captured. Allocating marks to online material will also help communicate the importance of specific material presented online.

Allocating a time slot in the class timetable for engaging with online material [38] may demonstrate the importance of asynchronous learning materials and help students who struggle with time management.

### Make full use of online tools and possibilities

Providing information in more than one format may help portray complex concepts as well as making material more engaging and accessible to different types of learners [51]. In the context of practical teaching, several case studies on one learning topic could be used; this would potentially highlight the diversity of patient populations and enable similar material to be presented in written, aural or visual formats without exact repetition.

Effective use of communication tools for staff and students may reduce feelings of social isolation and enable staff to gauge student engagement with online classes. For example, use of reaction features during online classes that enable students to provide feedback, for example, a digitalised ‘thumbs up’ may help communicate engagement and understanding. The online disinhibition effect [55] refers to the lack of restraint one feels in voicing thoughts and opinions online in comparison to in-person. The ability to ask questions anonymously may thus encourage students to interact with staff during live online classes.

### Seek feedback

Finding out what works well, and what does not work well, in relation to digitally enhanced practical skills teaching, from both the student and staff perspective, may enhance future implementation of technologically supported practical teaching. Furthermore, this may also help to facilitate positive attitudes and acceptability of digitally enhanced teaching approaches. Feedback detailing learner and staff experiences, as well as whether practical skill competencies are achieved, should be integrated into learning material and regularly reviewed.

## Discussion

This literature review aimed to provide a detailed summary of the barriers and opportunities presented by digitally enhanced practical skills teaching in health science education, and present recommendations to overcome these challenges. To the author’s knowledge, this is the first literature review exploring the barriers and opportunities presented by digitally enhanced practical skill teaching and learning in health science education. From the 29 papers included in this review, 7 barriers and 7 opportunities were identified through deductive thematic analysis. The barriers to digitally enhanced practical skills teaching identified are as follows: inaccessibility and inequity of online learning; digital illiteracy among staff; technological challenges; lack of engagement with preparatory material hinders practical learning; lack of staff–student interaction; negative attitudes towards online learning; skill suitability. The opportunities presented by digital technologies are; facilitates higher order learning; ability practice in a safe environment; efficacious use of class time; access to education; learning brought to life; diverse range of learning materials; promotes autonomous learning.

Perhaps one of the most significant findings of this review is the negative attitude held by many students towards online learning. When learning remotely during the Covid-19 pandemic, online practical skills teaching was perceived as an inadequate substitute for in-person clinical skills learning [29,31,43]. There are numerous operable suggestions this review recommends to combat the negative attitudes held towards digitally enhanced teaching.

Firstly, this review highlights the importance of continuous professional development, in the form of digital skills training, to improve instructor technological literacy and competency [56]. Ensuring educators have the skills to develop, design, and deliver high-quality resources will directly influence student interaction, engagement, and the overall student learning experience, enhancing student success. Institutional support is paramount in promoting positive attitudes towards the use of technology in teaching, and to ensure staff feel comfortable and confident utilising technology in their teaching practices [38]. IT training [27] and IT support [28] should therefore be prioritised among teaching staff and students to ensure an appropriate standard for teaching resources and methods to positively influence the learning experiences and decrease the negative perception many students hold towards online learning.

Secondly, it is important to explore the staff and student past-experiences of digitally enhanced practical skill teaching that may have played an influential role in developing negative attitudes. Understanding staff and student perspectives of what worked well and what didn’t work well when teaching and learning practical skills using technology will be useful in supporting its successful integration moving forwards as well as capitalising on the many opportunities it presents. Specifically, exploring student experiences and attitudes towards varied approaches (i.e. online, blended and face-to-face) to teaching practical skill will help educators make informed decisions on the acceptability and feasibility of digitally enhanced practical skill teaching moving forward.

Another important finding of this review is that not all skills are suited to online or digitally supported teaching. Haptic feedback can be difficult to mimic when teaching skills that require touch through demonstrative videos, with students struggling to refine technique in the absence of educator feedback [34]. However, there may be specific components of clinical skills that are appropriate to being taught using technology. There is therefore scope for investigating attributes of practical skills and their suitability to being taught and assessed with the support of digital technologies. It is important that as health science education continues to integrate digital technologies into practical skill teaching that educators can make evidence-based decisions on whether specific skills, or aspects of a skill, may be supported using digitally enhanced technologies. This is an important area that warrants further research.

Alongside the challenges identified in the literature, this review emphasises the unique opportunities presented to students when digitally enhanced teaching approaches are utilised in the classroom, or online, through blended learning. Using digital technology to supplement and support practical skills teaching provides students with opportunities not typically available to them in educational or clinical environments. Educators can develop realistic and diverse virtual patient simulations to provide students with a range of possible patients, facilitating immersive learning, critical thinking, and collaborative problem solving [37]. This further provides students with the ability to engage in repetitive practice and build self-efficacy, a practice often unavailable in busy clinical environments [51].

Students report greater confidence taking risks that can benefit their learning and making mistakes when engaging with virtual patients in a safe environment [48]. Digitised teaching tools, therefore, offer students a unique opportunity to move away from passive learning and become active learners [30,44]. However, some students struggle with adjusting to independent learning, requiring greater guidance and support from educators [39]. This finding may be explained by individual differences, or greater familiarity and experience with active learning for students of later years [6]; it may also be due to resource design quality, or limited opportunities for staff–student interaction [41]. Although exploring this finding in detail was beyond the scope of this review, recommendations for promoting a sense of community between those who teach and those who learn are provided. By asking students to have their cameras on, encouraging student feedback through digitised reactions (i.e. ‘thumbs up’ feature on Zoom), and engagement with discussion forums, staff can promote interconnectedness among classmates.

It is important to note, however, that not all the responsibility for the student experience can be placed on the educator. Improvements in teaching resources and learning experiences may also stem from greater collaboration between those designing and creating teaching tools and the users. Such collaboration could positively inform future technological development by taking into consideration what both educators and students need to enhance learning. For example, students emphasise the importance of immediate feedback in remote learning [10], and although the interactive components of MCQs are efficacious in ensuring knowledge acquisition [10], innovative and creative techniques are required to provide informative feedback on skill acquisition and competency when taught using digital technology.

The review’s findings should be considered alongside its limitations. Firstly, the included articles were heterogeneous in their methodological design that can be deemed as negatively influencing the quality of the review. However, including a wide range of methodological frameworks enabled an integrated approach to addressing the aim of the current literature review. Secondly, it is recommended that two or more researchers carry out an independent search strategy in parallel for quality assessment [57]; the absence of moderation in the screening process can be seen as a limitation of the methodological rigor of the review. Finally, it can be potentially seen as a limitation that the final themes are multifaceted with slight theme overlapping. For example, although negative attitudes and inaccessibility and inequity of online learning are two separate themes, there are interconnections; the researcher identified negative attitudes towards digital technologies can arise when students lack access to essential learning equipment. The researcher explored the meaning assigned to the data across varied contexts throughout data familiarisation and the coding process to differentiate conceptual theme patterns, leading to decisions on theme merging or distinction whereby independent themes are not absolutely discrete. One final limitation in this area of medical education is the divergent nature of educational design and delivery across the health sciences and across the globe. While an effort was made to ensure recommendations were general enough to apply to several contexts, it was also necessary to provide actionable examples. As such examples given purposefully vary from tasks which would require little resources and local effort to more capital projects requiring input from several stakeholders.

Despite its limitations, this literature review demonstrates the acceptability and usability of technology enhanced practical skills teaching in health science education among students and educators. Traditional teaching has become representative of the pre-internet era [6] while maximising the benefits of both online and offline teaching approaches in the form of blended learning is viewed as a lifelong approach to education [53]. However, this review highlights that implementing clinical skills teaching online poses practical challenges. Moving forward, it is therefore important to listen and learn from staff and student experiences, to acknowledge and address their concerns when it comes to digitally supported practical skill teaching.

## Conclusion

Educators and students were required to abruptly adapt to new distant teaching approaches during the Covid-19 pandemic. Although it may not be intuitive to consider practical skill teaching and learning suited to remote learning, teaching staff adopted innovative and creative techniques to support practical skill teaching using digital technologies. Health science education is therefore provided with a unique opportunity to learn from staff and student experiences and perceptions of practical skills teaching and learning before and during the Covid-19 pandemic. This review identifies and outlines the institution-level, staff-level, and student-level challenges to digitally enabled practical teaching, and recommendations are provided to overcome these barriers. This review also illustrates the opportunities presented by technological integration for health science education post-pandemic. To consolidate the positive disturbances from the Covid-19 pandemic, potential barriers to online delivery and student engagement must be acknowledged and addressed by relevant stakeholders. Future research may benefit from exploring the various components of a practical skill that are suited, or not suited, to online or distant learning.

## Supplementary Material

Supplemental Material
