# Supplementary material for: A Literature Review of Barriers and Opportunities Presented by Digitally Enhanced Practical Skill Teaching and Learning in Health Science Education
Source: Med Educ Online. 2022 Apr 21;27(1):2068210. doi: 10.1080/10872981.2022.2068210 (PMC9037199; doi:10.1080/10872981.2022.2068210)
Supplement: Supplemental Material [file ZMEO_A_2068210_SM8844.docx]

**Supplementary Materials**

1: Search strategy for PubMed using MeSH terms.

[ MeSH ("computer-assisted instruction" OR "education, distance") OR (Ti/Ab, "distance education" OR ''online learning'' OR ''video recording'' OR ''online systems'') ] AND [ MeSH ("education, medical" OR "allied health occupations" OR "schools, medical" OR ''heath education'' OR ''students, medical'') OR TI/AB, ("education, medical" OR "allied health occupations" OR "schools, medical" OR ''heath education'' OR ''students, medical'') ] AND [ MeSH, ‘'Clinical Competence'' OR ''attitude'') OR (TI/AB, ('clinical skills training'' OR ''practical skills'' OR ''clinical skills'')]
